# PDK-1 mediated Hippo–YAP–IRS2 signaling pathway and involved in the apoptosis of non-small cell lung cancer cells

**DOI:** 10.1042/BSR20182099

**Published:** 2019-05-14

**Authors:** Guofang Wang, Xiaomei Liu, Jiali Xie, Jinfei Meng, Xiaoqin Ni

**Affiliations:** 1 Respiratory Medicine, Yan’an University Affiliated Hospital, Yan’an 716000, China; 2 Nursing Department, Yan’an University Affiliated Hospital, Yan’an 716000, China

**Keywords:** Hippo-YAP, IRS2, Non-small cell lung cancer, PDK-1

## Abstract

Pyruvate dehydrogenase kinase-1 (PDK-1), a gatekeeper enzyme, was involved in cancer progression, such as tumor angiogenesis, cell survival, and growth. Recent evidence indicated that PDK-1 may be involved in lung cancer, however, the function and underlying mechanism of PDK-1 is remaining unclear. In the present study, our aim was to investigate the role and mechanisms of PDK-1 in human non-small cell lung cancer (NSCLC) cells. We first observed that PDK-1 was highly expressed in NSCLC cell lines. PDK-1 silence resulted in the inhibition of NSCLC cell survival. Also, cell apoptosis and caspase-3 activity were increased by PDK-1 knockdown in H1299 and A549 cells. Attenuation of PDK-1 expression blocked YAP and insulin receptor substrate 2 (IRS2) expression, and PDK-1 silence suppressed IRS2 expression dependent on Hippo–YAP signaling pathway. Moreover, further studies confirmed that YAP or IRS2 overexpression reversed the action of PDK-1 in NSCLC cells. In conclusion, our findings indicate that PDK1/Hippo–YAP/IRS2 signaling pathway plays a critical role in NSCLC cell survival and apoptosis.

## Introduction

Lung cancer, the most frequently diagnosed cancers, continues to be the leading cause of death in cancer all over the world [1]. Non-small cell lung cancer (NSCLC) accounts for approximately 85–90% of all new lung cancer cases [2]. Although standard treatment (platinum-based chemotherapy with maintenance therapy or without maintenance therapy and combined with second-line cytotoxic chemotherapy) for NSCLC was used, the median survival of patients with advanced NSCLC is approximately 1 year, thus the effective methods for treatment advanced NSCLC remains an urgent need [3,4]. Evidence also indicated that the cancer-related molecules play important roles in NSCLC [5,6]. Recently, evidence suggested that pyruvate dehydrogenase kinase-1 (PDK-1) implicated in lung cancer, however, the specific function and underlying mechanism of PDK-1 in NSCLC remains unknown.

Pyruvate dehydrogenase (PDH), a mitochondrial multienzyme complex, plays roles in the modulation of carbohydrate homeostasis and catalyses of the oxidative decarboxylation of pyruvate [7,8]. Pyruvate dehydrogenase kinases (PDKs) have four isomeric forms, including PDK-1, PDK-2, PDK-3, and PDK-4 [9]. The four PDK isoforms are distributed differently in tissues, PDK-1 is mostly expressed in heart, PDK-2 is abundant expressed in many tissues, PDK-3 is highly expressed in testis, and PDK-4 is mostly expressed in heart and skeletal muscle; moreover, PDK-3 and PDK-4 are lowly expressed in lung and kidney [10]. Among the four PDK isoforms, PDK-1 implicated into malignancy cancer [11]. Accumulating evidence indicated that the key enzyme PDK-1 was involved in metabolic reprogramming of tumors and was highly expressed in types of cancer, such as breast cancer [12], head and neck squamous cancer [13], myeloma [14], gastric cancer [15], and lung cancer [16].

Hippo pathway, an evolutionarily conserved pathway, implicated in diverse physiology and pathology processes, such as tissue size, tissue repair, tissue regeneration, and wound healing [17]. Hippo pathway also regulated many cellular processes, including cell differentiation, proliferation, and apoptosis [18]. The mammalian Hippo pathway has two downstream effectors, Yes-associated protein (Yap) and its paralog TAZ [19]. The transcription co-activator YAP could regulate tissue homeostasis, cell growth, and organ size [20]. It has been well documented that Hippo–YAP pathway was involved in the regulation of cancer cell biology, including cancer cell invasion and metastasis [21]. Moreover, another study has also demonstrated that the Hippo–YAP pathway plays important roles in the tumorigenesis and cancer development of lung tissues [22]. In the present study, we describe the role of the PDK-1 in regulating cell apoptosis in NSCLC cells. We confirmed that the PDK-1 is involved in NSCLC cell apoptosis, the underlying mechanism is related to Hippo–YAP/IRS2 signaling network.

## Materials and methods

### Cell culture

Human lung normal control cell line HBEC-3KT (HBEC) and human NSCLC cell lines (H1650, A549, H1299, H460, and H358) were all purchased from the ATCC (Manassas, VA, U.S.A.). All cells were cultured in RPMI-1640 medium, supplementary with fetal bovine serum (FBS, 10%), penicillin (100 units/ml), and streptomycin (100 mg/ml). Cells were maintained in an incubator containing 5% CO_2_ at 37°C.

### Cell transfection

PDK-1 siRNA and the siRNA control (siNC) were obtained from Dharmacon research (Lafayette, CO, U.S.A.). A549 siNC, H1299 siNC, A549 PDK-1 siRNA, and H1299 PDK-1 siRNA cells were generated by siRNA vectors specific for the control or PDK-1 vector by using lipofectamine 3000 (Thermo Fisher Scientific, Rockford, IL, U.S.A.) according to the manufacturer’s recommended conditions. PDK-1 siRNA and pcDNA3.1, PDK-1 siRNA and pcDNA3.1-YAP, PDK-1 siRNA and pcDNA3.1-IRS2 were co-transfected into A549 and H1299 cells by using lipofectamine 3000.

### MTT assay

For the cell survival assay, A549 and H1299 cell were plated in 96-well plate at a density of 5 × 10^5^ cells/well. Cells were transfected as mentioned above and cultured for 48 h. 20 μl (5 mg/ml) of MTT [3-(4,5-dimethylthiazol-2-yl)-2,5-diphenyl tetrazolium bromide] was added to each well and cultured for another 4 h at 37°C. DMSO was used to dissolve the MTT-formazan and the absorbance was determined at 570 nm.

### Cell apoptosis

For the apoptosis assay, A549 and H1299 cells were transfected and cultured as stated. Cell apoptosis was assayed by using an enzyme-linked immunosorbent assay (ELISA) for DNA fragments (Cell Death Detection ELISA assay, Roche, Mannheim, Germany) according to the methods described previously [23].

### Caspase-3

To further confirmed cell apoptosis, the caspase-3/7 activation was measured by using the Caspase-Glo 3/7 assay kit (Promega) as described previously [24].

### RT-PCR

The quantification of PDK-1 mRNA was detected by RT-PCR. Total RNAs were extracted by TRIzol RNA reagent (Invitrogen, Carlsbad, CA, U.S.A.). Total RNA (2 μg) was reverse transcribed to cDNA using High-Capacity cDNA ArchiveTM Kit according to the manufacturer’s protocols. TransStart Top Green qPCR SuperMix (TransGen Biotech, Beijing, China) was used for RT-PCR analyses. β-Actin was used as internal control.

### Western blot

Cellular proteins were extracted and equal amounts of protein fractionated by on 10% SDS-PAGE. The proteins were electrotransferred onto a PVDF membrane and blocked with 5% non-fat dry milk in PBST for 1 h. Antibodies specific for PDK-1 (ab110025, mouse monoclonal, 1:1000, Abcam, Cambridge, MA, U.S.A.), YAP (sc-376830, mouse monoclonal, 1:100, Santa Cruz Biotechnology, Santa Cruz, CA, U.S.A.), insulin receptor substrate 2 (IRS2) (ab134101, rabbit monoclonal, 1:1000, Abcam), and β-actin (ab6276, mouse monoclonal, 1:10000, Abcam) were used for immunoblot assays. The proteins were developed by an enhanced chemiluminescence (ECL) kit.

### Statistical analysis

Data in the present study are presented as the mean ± SD from the number of three independent experiments performed. Data were analyzed by using SPSS statistical software, version 21 (SPSS Inc., Chicago, IL, U.S.A.). Statistical significance was determined with ANOVA and two-tailed Student’s *t*-test. All experiments were repeated at least three times in the five replicate experiments. A value of *P*<0.05 was considered statistically significant.

## Results

### PDK-1 was increased in NSCLC cell lines

**Figure 1:**
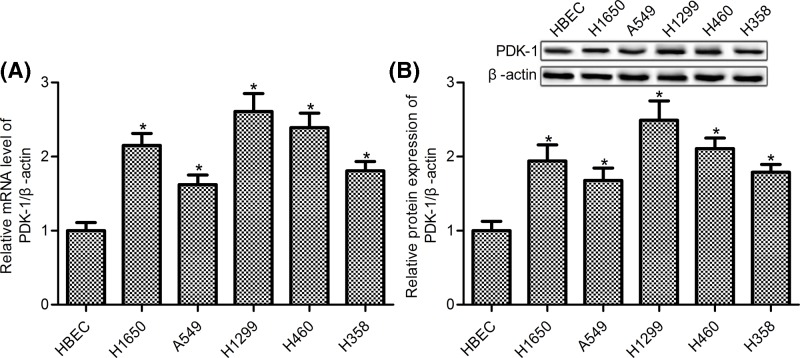
Increased PDK-1 expression in NSCLC cell lines (**A**) PDK-1 mRNA was detecting by RT-PCR; (**B**) Western blot was used to measure the protein expression of PDK-1. Data are presented as the mean ± SD from the number of three independent experiments performed. *P<0.05 vs. HBEC cells.

To assess the dysregulation of PDK-1 in NSCLC cells, we used five NSCLC cell lines including H1650, A549, H1299, H460, and H358 and the human lung normal control cell line HBEC-3KT (HBEC) in the present study. RT-PCR and Western blot assay showed that PDK-1 mRNA and protein expression levels were markedly elevated in all five NSCLC cell lines compared with the control HBEC cells (Figure 1, *P*<0.05). We observed that PDK-1 was highest in H1299 cells and lowest in A549 cells, we used these cells for our further studies.

### PDK-1 depletion decreased cell survival of NSCLC cells

**Figure 2:**
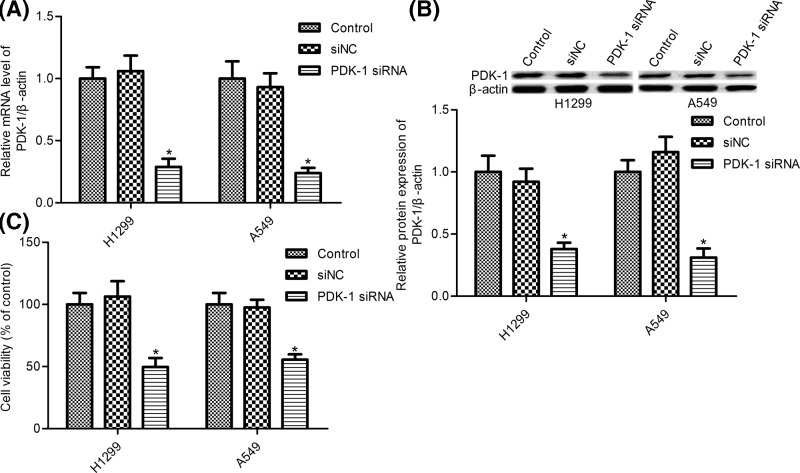
PDK-1 silence suppressed NSCLC cell survival (**A**) PDK-1 mRNA was detecting by RT-PCR; (**B**) Western blot was used to measure the protein expression of PDK-1; (**C**) cell survival was measured by MTT assay. H1299 cells and A549 cells were transfected with PDK-1 siRNA and the siRNA control (siNC) for 48 h. Data are presented as the mean ± SD from the number of three independent experiments performed. ^*^P<0.05 vs. control group.

To gain the function of PDK-1 in NSCLC cells, PDK-1 siRNA and siRNA control (siNC) transfected H1299 cells and A549 cells were constructed. RT-PCR and Western blot assay demonstrated that PDK-1 siRNA transfection significantly inhibited PDK-1 expression at mRNA and protein levels (Figure 2A,B). We first examined the effect of PDK-1 on H1299 cells and A549 cells survival, MTT assay showed that PDK-1 silence impeded cell survival both in H1299 cells and A549 cells compared with the siNC group (Figure 2C, *P*<0.05).

### PDK-1 depletion increased NSCLC cell apoptosis and caspase-3/7 activity

**Figure 3:**
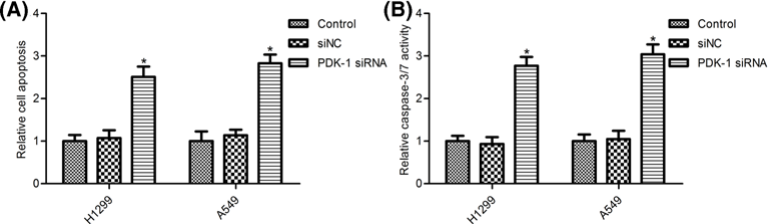
PDK-1 depletion promoted NSCLC cell apoptosis (**A**) Cell Death Detection ELISA assay was used to measure cell apoptosis; (**B**) Caspase-Glo 3/7 assay kit was used to measure caspase-3/7 activity. H1299 cells and A549 cells were transfected with PDK-1 siRNA and the siRNA control (siNC) for 48 h. Data are presented as the mean ± SD from the number of three independent experiments performed. ^*^P<0.05 vs. control group.

We next examined the effects of PDK-1 on H1299 cell and A549 cell apoptosis, as shown in Figure 3A, PDK-1 silence promoted H1299 and A549 cell apoptosis compared with the siNC group (*P*<0.05). To confirm these results, we performed caspase-3/7 activity experiments using Caspase-Glo 3/7 assay kit. We observed that, caspase-3/7 activity was markedly increased in PDK-1 depletion H1299 cells and A549 cells compared with the siNC group (as shown in Figure 3B, *P*<0.05).

### Down-regulation PDK-1 resulted in the low expression of Hippo–YAP/IRS2 signaling

**Figure 4:**
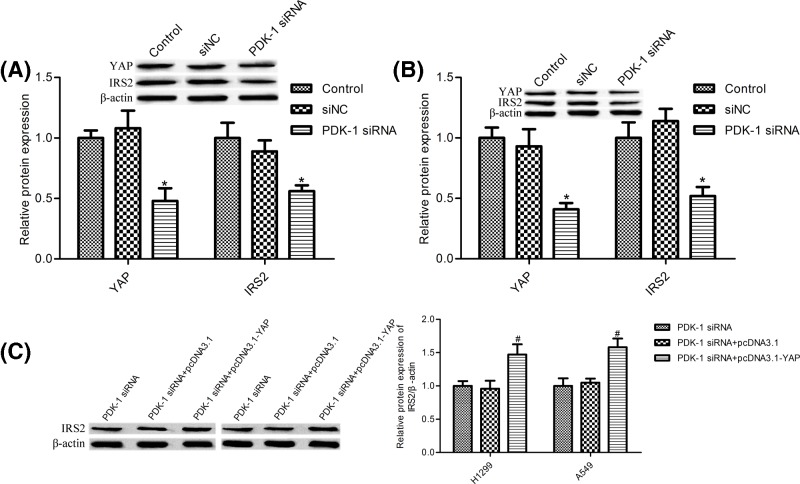
PDK-1 depletion suppressed Hippo–YAP/IRS2 signaling pathway (**A**) Western blot was used to measure the protein expression of YAP and IRS2 in H1299 cells; (**B**) YAP and IRS2 protein expression were measured in A549 cells by Western blot; (**C**) YAP overexpression markedly increased IRS2 protein expression in H1299 and A549 cells. H1299 and A549 cells were transfected with PDK-1 siRNA and the siRNA control (siNC) or co-transfected with PDK-1 siRNA and pcDNA3.1 or PDK-1 siRNA and pcDNA3.1-YAP for 48 h. Data are presented as the mean ± SD from the number of three independent experiments performed. ^*^P<0.05 vs. control group, ^#^p<0.05 vs. PDK-1 siRNA group.

In the previous reports, evidence suggested that PDK-1 can mediate Hippo–YAP pathway, moreover, YAP regulates IRS2 pathway [25,26]. In the present study, we observed that PDK-1 silence suppressed YAP and IRS2 protein expression both in H1299 cells and A549 cells (Figure 4A,B); also we demonstrated that YAP overexpression markedly increased IRS2 protein expression compared with PDK-1 silence group (Figure 4C), these results indicated that PDK-1 knockdown impeded IRS2 expression through inhibiting YAP expression.

### PDK-1 silence involved in NSCLC cell survival and apoptosis through Hippo–YAP/IRS2 signaling pathway

**Figure 5:**
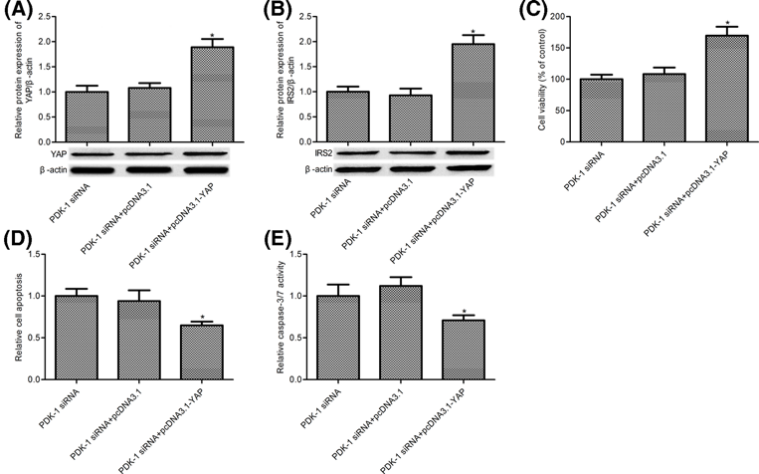
PDK-1 depletion promoted cell apoptosis through Hippo–YAP/IRS2 signaling pathway Western blot was used to measure the protein expression of (**A**) YAP and (**B**) IRS2; (**C**) cell survival was measured by MTT assay; (**D**) Cell Death Detection ELISA assay was used to measure cell apoptosis; (**E**) Caspase-Glo 3/7 assay kit was used to measure caspase-3/7 activity. H1299 cells were co-transfected with PDK-1 siRNA and pcDNA3.1 or PDK-1 siRNA and pcDNA3.1-YAP or PDK-1 siRNA and pcDNA3.1-IRS2 for 48 h. Data are presented as the mean ± SD from the number of three independent experiments performed. ^*^P<0.05 vs. PDK-1 siRNA group.

To further studied the molecular mechanism, PDK-1 siRNA and pcDNA3.1, PDK-1 siRNA and pcDNA3.1-YAP, PDK-1 siRNA and pcDNA3.1-IRS2 were co-transfected into H1299 cells. Results from Western blot showed that PDK-1 siRNA and pcDNA3.1-YAP, PDK-1 siRNA and pcDNA3.1-IRS2 co-transfection significant up- regulated the expression of YAP and IRS2 (Figure 5A,B, *P*<0.05). Evidence suggested that YAP or IRS2 overexpression significantly increased H1299 cell survival compared with PDK-1 siRNA group (as shown in Figure 5C, *P*<0.05). Further study demonstrated that YAP or IRS2 overexpression reversed the effects of PDK-1 siRNA on H1299 cell apoptosis and caspase-3 activity (as shown in Figure 5D,E, *P*<0.05).

## Discussion

Lung cancer is the most frequent cause of cancer-related death all over the world, and the 5-year survival rate is less than 15% [27]. Cancer cell proliferation and apoptosis involved in cancer development. In the present study, we observed that PDK-1 is highly expressed in NSCLC cell lines; PDK-1 depletion promoted cancer cell apoptosis and inhibited cell proliferation through Hippo–YAP/IRS2 signal pathway.

PDK-1 is a Ser/Thr kinase enzyme which involved in the inactivation of mitochondrial PDH by phosphorylation by using adenosine triphosphate (ATP) [28]. It has been reported that PDKs were significantly overexpressed in many types of human tumor samples [11]. Baumunk et al. indicated that PDK-1 up-regulation is an early event of renal cell carcinoma development, however, PDK-1 is almost uncorrelated with the progression toward an aggressive phenotype [29]. Hur and his colleagues suggested that the high expression of PDK-1 might act as a biomarker of poor prognosis in gastric cancer patients and PDK-1 inhibitors may be a potential therapeutic target of the patients [15]. Also, Fujiwara et al. reported that PDK-1 serve as a biomarker in multiple myeloma of poor prognosis and chemicals inhibiting PDK-1 may be take part in cancer therapy [14]. Liu and Yin indicated that PDK-1 was highly expressed in lung cancer tissue, and promotes lung cancer cell proliferation, migration and Warburg effect in lung cancer [30]. Although the effects of PDK-1 on lung cancer cell biology have been reported, the molecular mechanism is still unknown. In agreement with the previous reports, our study indicated that PDK-1 was highly expressed in NSCLC cell lines compared with the control cells. Our study indicated that PDK-1 silence promoted cancer cell apoptosis and caspase-3 activity.

YAP is one of the mammalian Hippo-pathway effector [31], and evidence showed that YAP is an effective regulator of cell proliferation and apoptosis [32,33]. Also, YAP has been associated with cancer development, including squamous cell carcinoma [34], brain tumor [35], and colorectal cancer [36]. A recent study found that Hippo–YAP signaling pathway plays important roles in CXCR4 depletion mediated epithelial mesenchymal transition of NSCLC [37]. In the present study, we focused on whether Hippo–YAP signaling pathway is associated with the effects of PDK-1 silence on NSCLC cell apoptosis. We observed that PDK-1 silence markedly suppressed YAP expression, and re-expressed YAP by pcDN3.1-YAP vector reversed the effects of PDK-1 on NSCLC cell apoptosis. Interestingly, Jeong et al. confirmed that YAP/TAZ activation could up-regulated the IRS2 expression and then amplified AKT signaling [38]. In the present study, we found that PDK-1 silence obviously impeded the expression of IRS2 through inhibiting the level of YAP. Overexpression IRS2 mitigated PDK-1 silence induced cancer cell apoptosis.

In summary, we demonstrate that PDK-1 knockdown induced cancer cell apoptosis may be through Hippo–YAP/IRS2 signal pathway. This inhibition of the YAP/IRS2 pathway by PDK-1 silence can, at least in part, explain the molecular mechanism of PDK-1 depletion in NSCLC cell apoptosis.

## Abbreviations

ELISA: enzyme-linked immunosorbent assay
IRS2: insulin receptor substrate 2
MTT: 3-(4,5-dimethylthiazol-2-yl)-2,5-diphenyl tetrazolium bromide
NSCLC: non-small cell lung cancer
PDH: pyruvate dehydrogenase
PDK-1: pyruvate dehydrogenase kinase-1
